# Chemical hazards in smoked meat and fish

**DOI:** 10.1002/fsn3.2633

**Published:** 2021-10-18

**Authors:** Ogouyôm Herbert Iko Afé, Yénoukounmè Euloge Kpoclou, Caroline Douny, Victor Bienvenu Anihouvi, Ahmed Igout, Jacques Mahillon, Djidjoho Joseph Hounhouigan, Marie‐Louise Scippo

**Affiliations:** ^1^ Laboratory of Food Analysis Department of Food Sciences Faculty of Veterinary Medicine Fundamental and Applied Research for Animals & Health (FARAH) Veterinary Public Health University of Liège Liège Belgium; ^2^ Laboratory of Food Sciences School of Nutrition and Food Sciences and Technology Faculty of Agronomic Sciences University of Abomey‐Calavi Cotonou Benin; ^3^ Department of biomedical and preclinical Sciences Faculty of Medicine University of Liège Liège Belgium; ^4^ Laboratory of Food and Environmental Microbiology Faculty of Bioscience Engineering UCLouvain Louvain‐la‐Neuve Belgium

**Keywords:** benzo(a)pyrene, food safety, heat‐induced compounds, nitrosamines, systematic review

## Abstract

This review aims to give an insight into the main hazards currently found in smoked meat and fish products. Literature research was carried out on international databases such as Access to Global Online Research in Agriculture (AGORA) database, Science direct, and Google scholar to collect and select 92 relevant publications included in this review. The smoking process was described and five hazards mostly found in smoked fish and meat were presented. The heat‐induced compounds such as polycyclic aromatic hydrocarbons, heterocyclic amines, and nitrosamines were found in smoked fish and meat. Other hazards such as biogenic amines and heavy metals were also present in smoked fish and meat. The levels of these hazards reported from the literature exceeded the maximal limits of European Union. A brief description of risk assessment methodology applicable to such toxic compounds and risk assessment examples was also presented in this review. As most of the hazards reported in this review are toxic and even carcinogenic to humans, actions should be addressed to reduce their presence in food to protect consumer health and to prevent public health issue.

## Graphical abstract

‐Main hazards reported in smoked meat and fish are toxic and carcinogenic to humans

‐Levels of chemical hazards reported from literature exceeded the maximal limits of European Union

‐Consumption of smoked meat and fish presents risk for consumers ‘health

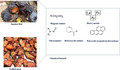

## 1 INTRODUCTION

Fish and meat preservation is a big challenge in different regions of the world. Among fish preservation methods, smoking is the mostly used method (Berkel et al., 2005; Nout et al., 2003; Toth & Potthast, 1984). It extends shelf life and confers special taste and aroma to the end products (Igwegbe et al., 2015; Yusuf et al., 2015). Despite these advantages, the consumption of smoked fish or meat products presents health concern due to process contaminants. Several authors reported acrolein, acrylamide, furan, heterocyclic amines, monochloropropanediol (MCPD), nitrosamine, and polycyclic aromatic hydrocarbons (PAHs) as heat‐induced toxic compounds in foods and dealt with their risk assessment (Akpambang et al., 2009; Alomirah et al., 2011; Domingo & Nadal, 2015; Larsen, 2006; Mey et al., 2014; Skog, Johansson, & Jaègerstad, 1998; Stadler & Lineback, 2009; Swann, 1977; Yurchenko & Molder, 2007). Among these various heat‐induced compounds, PAHs and heterocyclic amines are mainly associated with smoking or grilling process. Moreover, due to the amino acid composition of fish and meat, some toxic compounds like biogenic amines and even nitrosamines may be formed. Other environmental hazards like heavy metals can also be found in fish and meat. The consumption of food contaminated with these compounds could result in adverse effects on human health including cancer (EFSA (European Food Safety Authority), 2008; EFSA (European Food Safety Authority), 2011). This literature review aims to focus on chemical hazards (nitrosamines, heterocyclic amines, PAHs, heavy metals, and biogenic amines) commonly reported in smoked fish and meat.

## 2 METHODOLOGY

Publications included in this review were from international databases such as Access to Global Online Research in Agriculture (AGORA) database, Science direct, and google scholar. Original and review papers were collected on December 2019 and updated on July 2021 using key words such as “smoking,” “heterocyclic amines,” “PAHs,” “benzo(a)pyrene (BaP),” “heavy metals,” “nitrosamine,” “biogenic amines,” “histamine,” “risk assessment,” “fish,” and “meat.” Only 112 relevant papers in accordance with the topic of this review were included on the basis of their keywords. The software Endnote, version 13 was used to manage and rank the collected publications in different subgroups according to each topic.

## 3 RESULTS

### 3.1 Smoking process

Smoking is a food processing method used as a preservation method to extend shelf life of food by reducing moisture content and microorganism load (Köse, 2010). Smoking is also used to improve sensorial characteristics including taste, aroma, and appearance of smoked fish and meat (Berkel et al., 2005; Codex Alimentarius, 2009). Two types of smoking processes are commonly used. The “cold” smoking process in which the temperature of the product does not exceed 30°C and “hot” smoking process during which food such as fish is well cooked and temperature in the center of the product may reach up to 60–85°C (Berkel et al., 2005; Stołyhwo & Sikorski, 2005). According to Berkel et al. (2005), there is a third smoking process called smoke‐drying which is hot smoking followed by a drying step carried out in the smoking equipment. Hot smoking and smoke‐drying are frequently used to preserve fish in African countries (Assogba et al., 2019). According to Codex Alimentarius (2013), the smoke‐drying process enables us to obtain dried products with a water activity lower or equal to 0.75, allowing keeping the end product at room temperature and to control bacterial and fungi alteration.

**FIGURE 1:**
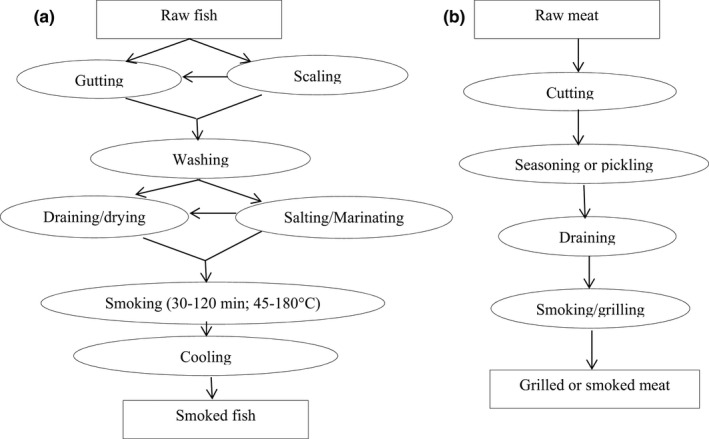
(a) Flow chart of smoked fish production (Adeyemi et al., 2013; Adeyeye et al., 2015; Assogba et al., 2019; Dègnon et al., 2013; Goulas & Kontominas, 2005; Ubwa et al., 2015) and (b) smoked meat production (Poligné et al., 2001; Roseiro et al., 2011)

During smoking, fish or meat and their products are submitted directly or indirectly to smoke produced by partial burning of wood. Direct smoking is a process during which fish or meat is laid on mesh trays above the embers, whereas in indirect smoking, smoke is produced in a separate chamber and fish is smoked in another chamber (Codex Alimentarius, 2009). Traditional fish smoking is carried out in kilns (barrel of locally made clay) using fuel such as wood, charcoal, wood sawdust, wood chips, bagasse, corn cobs, coconut husks, and shells (Assogba et al., 2019; Codex Alimentarius, 2009; Kpoclou et al., 2014; Stołyhwo & Sikorski, 2005). Smoke is composed of a mixture of about 380 compounds, mainly phenols, aldehydes, ketones, organic acids, alcohols, esters, hydrocarbons, and various heterocyclic compounds (Codex Alimentarius, 2009; Toth & Potthast, 1984). Some of them such as phenolic, carbonyls, furan derivatives, organic acids, and their esters affect the sensory quality, but could also improve the shelf life of the product by inhibiting the growth of spoilage bacteria (Ciecierska & Obiedzinski, 2007; Gomez‐Guillén et al., 2009; Igwegbe et al., 2015; Stołyhwo & Sikorski, 2005; Yusuf et al., 2015). However, carcinogenic compounds such as PAHs, nitrosamines, and heterocyclic amines may be formed during the smoking process either from pyrolysis of organic matter and transferred inside the food or directly produced inside the food as a result of reactions between food composition and heat (Skog et al., 1998; Stołyhwo & Sikorski, 2005; Viegas et al., 2012; Yurchenko & Molder, 2007). Figure 1 shows different smoking methods of fish and meat reported from the literature.

### 3.2 Nitrosamine, heterocyclic amines, and polycyclic aromatic hydrocarbons

#### 3.2.1 Nitrosamines

**FIGURE 2:**
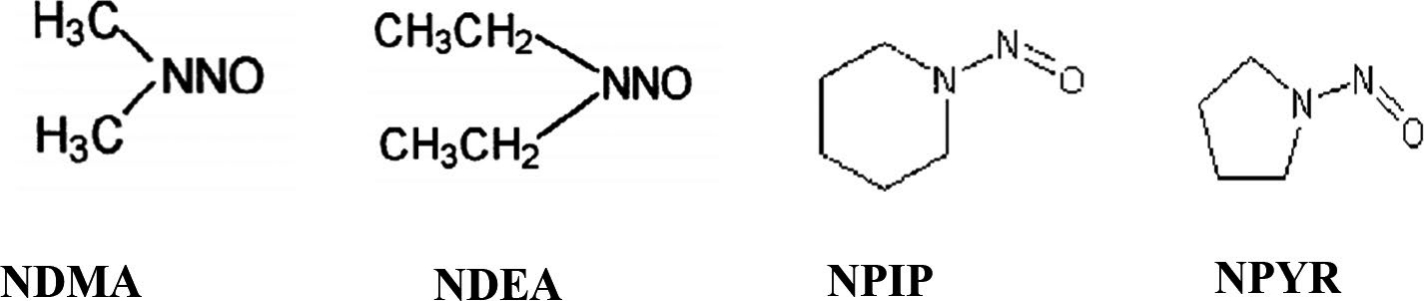
Chemical structure of four examples of nitrosamines: N‐nitrosodiméthylamine (NDMA); N‐nitrosodiethylnitrosoamine (NDEA); 1‐nitrosopiperidine (NPIP) and 1‐nitrosopyrrolidine (NPYR) (PubChem, 2020).

Nitrosamines (Figure 2) or N‐nitroso compounds (N‐nitrosodiméthylamine (NDMA), N‐nitrosométhyléthylamine (NMEA), N‐nitrosodiethylnitrosoamine (NDEA), Nitrosodipropylamine (NDPA), N‐nitrosodibutylamine (NDBA), N‐nitrosomorpholine (NMOR), 1‐nitrosopiperidine (NPIP), 1‐nitrosopyrrolidine (NPYR), N‐nitrosodiéthanolamine (NDELA), 1‐methyl‐3‐nitro‐1‐nitrosoguanidine (MNNG), N‐nitroso‐N‐ethylbutylamine (NEBA), N'‐nitrosoanabasine (NAB), and 4‐(N‐nitrosomethylamino)‐1‐(3‐pyridyl)‐1‐butanone (NNK)) are a group of toxic compounds produced mainly in meat products during heat processing (Belitz et al., 2009; Domanska & Kowalski, 2003; Reinik, 2007).

N‐nitroso compounds can be subdivided into two groups (Herrmann et al., 2015): volatile nitrosamines (NDMA, NMOR, NMEA, NPYR, NDEA, and NPIP) and nonvolatile nitrosamines (N‐nitrososarcosine (NSAR), N‐nitrosoproline (NPRO), N‐nitrosomethylaniline (NMA), N‐nitroso‐thiazolidine‐4‐carboxylic (NTCA) acid, and N‐nitroso‐2‐methylathiazolidine‐4‐carboxylic acid (NMTCA)).

The human exposure to N‐nitroso compounds is from environment, tobacco smoke, and the diet which has been identified to be the main source (Jakszyn et al., 2005). N‐nitroso compounds formation requires substrates (primary amine, secondary amine, tertiary amine, amides, secondary amino acids, quaternary ammonium salts, etc.) and a nitrosating agent (nitrite, nitrates, and nitrogen oxides) through several reactions (Filho et al., 2003; INERIS (Institut National de l'Environnement Industriel et des RISques), 2014; Reinik, 2007). Nitrogen oxides are formed either from the addition of nitrate and/or nitrite to foods or from the heating process of food such as smoking, during which nitrogen molecular can be oxidized or present in the smoke (INERIS (Institut National de l'Environnement Industriel et des RISques), 2014; Jakszyn et al., 2005). Al Bulushi et al. (2009) reported NPYR and NPIP in vitro formation at high temperature (160°C, 2 h). Microorganisms (*Aspergillus* sp.; *Pseudomonas* sp.; *P. stutzeri; E*. *coli*) can be involved in N‐nitrosamine formation by reducing nitrates to nitrites, by degrading proteins to amines and amino acids, or by producing enzymes working at a suitable pH (2–4) for nitrosation (Al Bulushi et al., 2009; Ayanaba & Alexander, 1973; Drabik‐Markiewicz et al., 2009; Jägerstad & Skog, 2005; Jägerstad et al., 1998; Mills & Alexander, 1976; Rostkowska et al., 1998; Yurchenko & Molder, 2007). Nitrite and nitrate are frequently used in meat preservation and lead to nitrosamines formation due to reaction with amino compounds either in the stomach or within the food product (Filho et al., 2003; Pan et al., 2011; Sebranek & Bacus, 2007; Swann, 1977). It is the case of meat products such as sausages, *ham*, and *salami* where the addition of nitrite and nitrate was used to inhibit the formation of spoilage bacteria (Drabik‐Markiewicz et al., 2009; Filho et al., 2003; Hustad et al., 1973). The nitrosamines are found in smoked meat, grilled meat, canned meat, and pickled meat at different levels (Table 1), but not in raw meat where there is not enough nitrite and amines for its production (Yurchenko & Molder, 2007). Studies carried out on nitrosamine determination in fish products mostly focused on NDMA determination because of its precursor dimethylamine (DMA) which is widely formed in marine fish (Al Bulushi et al., 2009). NDMA is classified in Group 2A (probably carcinogenic to humans) by the International Agency for Research on Cancer (IARC (International Agency for Research on Cancer), 2010), whereas N‐nitrosonornicotine (NNN) and 4‐(N‐nitrosomethylamino)‐1‐(3‐pyridyl)‐1‐butanone (NNK) are classified in Group 1 (carcinogenic to humans). Belitz et al. (2009) reported NDMA in cured meat processed with pickling with levels ranging between 0.5 and 15 μg/kg (Table 1). Herrmann et al. (2014) also reported NDMA in smoked pork fillet (1.3 µg/kg) and smoked ham (2.1 µg/kg).

**TABLE 1:** Concentrations of volatile N‐nitrosamines in various smoked or grilled fish and meat as reported from the literature

Processing	*N*‐nitrosamines (µg/kg)						References
	NDMA	NDEA	NPYR	NPIP	NDBA	NDPA	
Smoked meat	0.2–1.4	0.3–0.7	0.2–19.5	0.4–2.3	0.4–0.9	0.3	Al‐Kaseem et al. (2014); Reinik (2007); Yurchenko and Molder (2007)
Cured meat (pickling salt)	0.5–15	nd	3.2–4.2	nd	Nd	nd	Belitz et al. (2009)
Grilled meat	0.2–3.2	0.3–0.6	0.8–14.6	1.0–2.8	0.2–0.4	˂0.1–0.3	Al‐Kaseem et al. (2014); Reinik (2007); Yurchenko and Molder (2007)
Smoked fish	˂0.1–2.8	˂0.1–0.5	0.4–25.4	˂0.2–7.8	˂0.2–6.0	nd	Reinik (2007)
Smoked chicken	1.2–2.1	˂0.1–0.3	0.5–22.1	˂0.1–5.3	0.1–6.3	nd	

Abbreviations: nd, not determined;NDBA, N‐nitrosodibuthylnitrosamine; NDEA, N‐nitrosodiethylnitrosamine; NDMA, N‐nitrosodimethylnitrosamine; NDPA, N‐nitrosodipropylamine; NPIP, N‐nitrosopiperidine; NPYR, N‐nitrosopyrrolidine.

Different analytical methods were used to analyze nitrosamines. The method of gas chromatography and mass spectrometry detection with ion monitoring using different columns has been used by several authors to identify and quantify nitrosamines (Filho et al., 2003; Herrmann et al., 2014; Swann et al., 1977; Yurchenko & Molder, 2007). However, only Thermal Energy Analyzer (TEA) detection is recognized as specifically for nitrosamines but expensive (Filho et al., 2003). Filho et al. (2003) developed methods for nitrosamine compounds analysis (extraction, preconcentration, and analysis) which allowed their determination even at trace levels. The separation of nitrosamines was performed using micellar electrokinetic chromatography and confirmation was achieved using gas chromatography coupled with mass spectrometry detection (Filho et al., 2003; Herrmann et al., 2014).

#### 3.2.2 Heterocyclic amines

**FIGURE 3:**
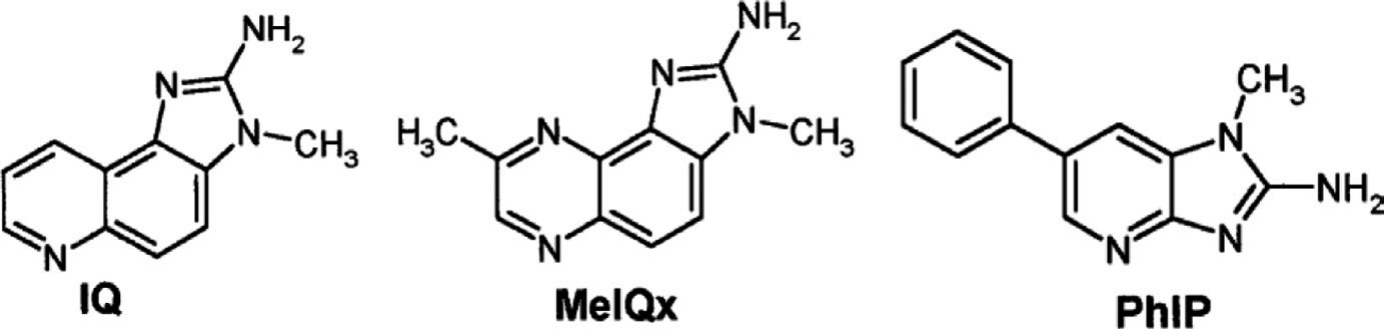
Chemical structure of three examples of heterocyclic amines: IQ ((2‐Amino‐3,4‐dimethylimidazo[4,5‐f]quinolone)); MeIQx (2‐amino‐3,8‐dimethylimidazo[4,5‐f]quinoxaline ) and PhIP (2‐amino‐1‐methyl‐6‐phenylimidazo[4,5‐b]pyridine) (PubChem, 2020)

Heterocyclic amines are toxic compounds produced in meat and fish during processing at temperature over 150°C (Haskaraca et al., 2014; Jägerstad & Skog, 2005; Puangsombat et al., 2011; Sinha et al., 1998; Solyakov & Skog, 2002). According to their chemical structures, two groups of heterocyclic amines can be distinguished: pyrolytic heterocyclic amines also known as amino‐carboline heterocyclic amines and thermic heterocyclic amines composed of imidazo‐quinolines (e.g., IQ ((2‐Amino‐3,4‐dimethylimidazo[4,5‐f]quinolone)), imidazoquinoxalines (e.g., MeIQx (MeIQx (2‐Amino‐3,8‐dimethylimidazo[4,5‐f]quinoxaline)), and imidazopyridines (e.g., PhIP (2‐Amino‐1‐methyl‐6‐phenylimidazo[4,5‐b]pyridine)) (Jägerstad & Skog, 2005; Viegas, Novo, Pinto, et al., 2012). The imidazo‐quinolines, imidazoquinoxalines, and imidazopyridines are three groups of precursors present in raw meat and fish muscle and could be produced from creatine or creatinine, free amino acids, and sugars through the Maillard reaction (Jägerstad & Skog, 2005; Viegas, Novo, Pinto, et al., 2012). The IARC (International Agency for Research on Cancer) classified MeIQx, MeIQ, and PhiP as possibly carcinogenic to humans (Group 2B). The 2‐amino‐3,8‐dimethylimidazo[4,5‐f]quinoxaline (8‐MeIQx) and 2‐amino‐1‐methyl‐6‐ phenylimidazo[4,5‐b]pyridine (PhIP) (Figure 3) are the most abundant heterocyclic amines formed in grilled beef, bacon, fish, and poultry (Turesky, 2007). Skog et al. (1998) reported the presence of heterocyclic amines in smoked fish and fried meat products. The authors showed that the use of wood charcoal induced high production of heterocyclic amines (1.6–4 ng/g MeIQx; 1.5–7.8 ng/g PhIP) contrary to coconut charcoal (0.7–1 ng/g MeIQx; 0.9–3 ng/g PhIP) (data not shown) in grilled salmon and beef samples (Viegas, Novo, Pinto, et al., 2012). Gibis (2016) reported high temperature (180°C and 220°C) and duration as key factors of heterocyclic amines production, mainly IQ, MeIQ, MeIQx, 4,8‐DiMeIQx, and PhIP. Table 2 shows different concentrations of some heterocyclic amines reported from the literature. Very few studies reported the presence of IQ in grilled or smoked foods. Levels of 1.6–2 ng/g were reported in grilled beef (Table 2). However, MeIQx was reported in several foods such as processed bacon and pork (Sinha et al., 1998) with levels ranging from 0.4 to 5.4 ng/g (Table 2). High levels of PhIP (till 480 ng/g) were reported from the literature (Table 2). Even though no maximal limit of heterocyclic amines was reported in the literature, their presence in food is a health concern and adequate food preparation procedures should be implemented having the ALARA (ALARA = as low as reasonably achievable) principle in mind. Lu et al. (2018) reported that the use of different spices (Garlic, onion, red chili, paprika, black pepper, and ginger) before deep‐frying of beef and chicken had inhibitory effects (43%–87%) on the formation of heterocyclic amines (data not shown).

**TABLE 2:** Concentration of main heterocyclic amines in cooked meat, as reported from the literature

Products	Cooking methods.	Heterocyclic amines (µg/kg)					References
		MeIQx	DiMeIQx	PhIP	IQ	MeIQ	
Bacon	Pan‐fried	0.4–4.3	nd	0.7–4.8	nd	nd	Sinha et al. (1998)
	Oven‐broiled	1.5–4	nd	1.4–30.3	nd	nd	
	microwaved	0.4–1.5	nd	3.1	nd	nd	
	Grilled	1.0–27	nd−9.3	nd−36	nd	nd	Knize et al. (1997); Skog et al. (1998).
Pork	Pan‐fried	0.4–5.4	nd	0.1–2.3	nd	nd	Sinha et al. (1998)
	Oven‐broiled	nd	nd	nd	nd	nd	
Beef	Grilled	0.5–6	0.1–1.2	0.6–27	0.2	nd	Fay et al., (1997); Murray and Lynch (1993); Wakabayashi et al. (1993);
	Barbecued	4.4	2.7	38	1.6	nd	Skog et al. (1998)
Chicken	Barbecued	0.3–9	0.1–3.1	27–480	nd	nd	Knize et al. (1996); Murray and Lynch (1993); Sinha et al. (1995)
	Grilled	0.6–2.3	0.5–3.1	21–315	nd	nd	Knize, Salmon, Hopmans, et al. (1997); Knize et al. (1997); Wakabayashi et al. (1993)

Abbreviations: nd, not determined; MeIQx = 2‐amino‐3,8‐dimethylimidazo[4,5‐f] quinoxaline; DiMeIQx = 2‐amino‐3,4,8‐trimethylimidazo[4,5‐f]quinoxaline; PhIP = 2‐amino‐1‐methyl‐6‐phenylimidazo[4,5‐b]pyridine; IQ = 2‐amino‐3‐methylimidazo[4,5‐f ]quinoline; MeIQ = 2‐amino‐3,4‐dimethylimidazo[4,5‐f]quinoline.

Heterocyclic amine determination was performed according to methods including extraction, purification injection, and quantification using high‐performance liquid chromatography coupled with diode array and fluorescence detectors (HPLC‐DAD/FLD) (Melo et al., 2008). Heterocyclic amines can also be extracted by solid‐phase extraction and analyzed by reverse phase HPLC or LC/MS (Oz & Yuze, 2016; Santos et al., 2004; Sinha et al., 1998; Viegas, Novo, Pinto, et al., 2012).

#### 3.2.3 Polycyclic aromatic hydrocarbons

Polycyclic aromatic hydrocarbons are toxic compounds having a low solubility in water and constitute a large class of organic compounds, containing 2 or more fused aromatic rings composed of carbon and hydrogen atoms (EFSA (European Food Safety Authority), 2008; SCF (Scientific Committee on Food), 2002). They are produced from incomplete combustion of the organic matter when foods such as fish or meat are processed by smoking, grilling, or roasting (Battisti et al., 2015; EFSA (European Food Safety Authority), 2008; Ingenbleek et al., 2019; Yusuf et al., 2015). Several studies reported that fat dropping in the flame during grilling processing contributes to PAHs formation (Chen et al., 2013; Viegas, Novo, Pinto, et al., 2012). Additionally, studies showed that PAHs formation depends on the type of raw material, smoking methods, fuel and kiln type, smoke composition and degree of exposure to smoke, and combustion temperature (Chen et al., 2013; Codex Alimentarius, 2009; Kpoclou et al., 2014; Stołyhwo & Sikorski, 2005). Traditional smoking or grilling is responsible for the production of high amounts of PAH in meat and fish as reported by Forsberg et al. (2012); Onyango et al. (2012); Iko Afé et al. (2020); Ubwa et al. (2015).

**FIGURE 4:**
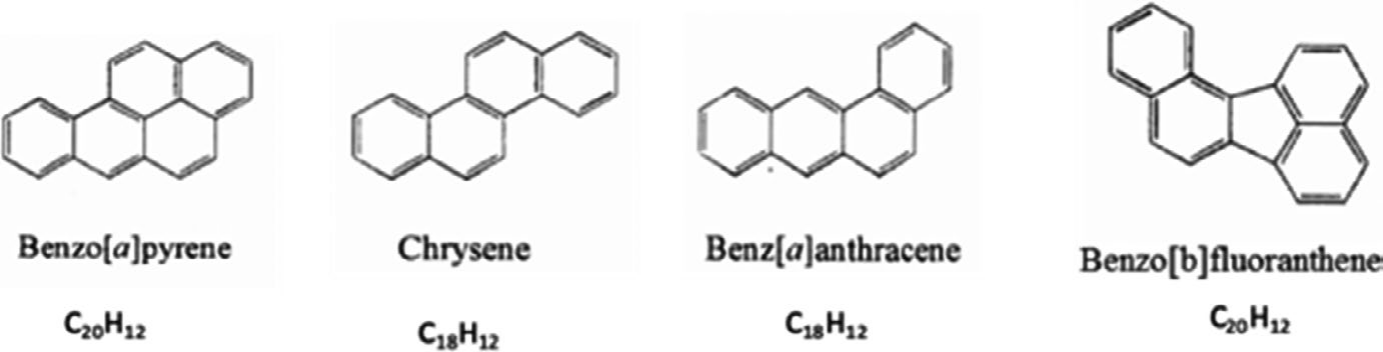
Chemical structure of the PAH4 for which a maximum limit in food has been set in EU (PubChem, 2020).

Consumers are exposed to PAHs according to three possible ways: by inhalation, contact with the skin, and consumption of contaminated food (EFSA (European Food Safety Authority), 2008; Silva et al., 2011). Likewise, foods are contaminated with PAHs either by environment (exhaust fumes of the engines, bush fires, etc.) or by traditional food processing (drying, smoking, grilling, etc.) (ANSES (Agence nationale de sécurité sanitaire de l’alimentation, de l’environnement et du travail), 2011). The main route of human exposure to PAHs is diet (ANSES (Agence nationale de sécurité sanitaire de l’alimentation, de l’environnement et du travail), 2011; EFSA (European Food Safety Authority), 2008). PAHs are genotoxic, carcinogenic, and mutagen (EFSA (European Food Safety Authority), 2008; SCF (Scientific Committee on Food), 2002). Due to their genotoxicity, sixteen PAHs have been included in a priority list of the European Union (EU) (SCF (Scientific Committee on Food), 2002). Among these 16 priority EU PAHs, benzo[a]anthracene (BaA), chrysene (CHR), benzo[a]pyrene (BaP), and benzo[b]fluoranthene (BbF) are four PAHs (named PAH4) (Figure 4) relevant in food due to their toxicity and occurrence (EFSA (European Food Safety Authority), 2008). PAHs are metabolized in the liver by cytochrome P450 (CYP1A1 in particular) into compounds named epoxides, which are able to bind to macromolecules such as proteins and nucleic acids (EFSA (European Food Safety Authority), 2008). After ingestion, before to reach the liver, PAHs come in contact with the intestinal microbiota, which can also have a metabolization role. Van de Wiele et al. (2005) evaluated the possible ways of biotransformation of PAHs in the human intestine using a simulator of the human intestinal microbial ecosystem (SHIME). These authors showed that PAHs are bioactivated in colon digestion into estrogenic metabolites, whereas the digestion of the stomach and small intestine does not generate any estrogenic metabolite. Moreover, the inactivation of the colon microbiote eliminated these estrogenic effects, which suggests that the estrogenic activity would be related to the bio‐activation of PAHs by the microbiote of the colon (Van de Wiele & Al, 2005). In addition to be carcinogenic, PAHs can thus be qualified of endocrine disrupters.

Among PAHs, BaP is the mostly used one for in *vivo* toxicological studies. After giving female mice BaP at doses >10 mg/kg b.w. (body weight) per day, impaired fertility was observed in their offspring. The studies on carcinogenicity of PAHs showed that the type of cancer developed after PAHs exposure depends on the exposure way. Indeed, a dermic exposure would induce tumors on the skin, whereas an exposure by oral way would induce gastric tumors. After oral exposure of laboratory animals to BaP, gastrointestinal tract, liver, and lung tumors were reported (EFSA (European Food Safety Authority), 2008). After feeding female mice with diets containing BaP at a concentration of 0, 5, 25, or 100 mg/kg of diet for 2 years, papillomas and carcinomas were observed in the forestomach, oesophagus, and tongue (Culp et al., 1998). Several authors also associate colorectal cancer with meat consumption and some of them established colorectal cancer (Gunter et al., 2007; Ronco et al., 2011; Sinha et al., 2005). Sinha et al. (2005) reported an increased risk of colorectal adenomas resulting from high BaP intake from both meat consumption and other food sources.

Benzo(a)pyrene is classified as carcinogenic to humans (Group 1), and CHR, BaA, and BbF are classified as possibly carcinogenic to humans (Group 2B) (IARC (International Agency for Research on Cancer), 2010).

The European commission set maximum levels of 2 and 12 μg/kg for benzo(a)pyrene (BaP) and the PAH4, respectively, in smoked meat and smoked fish products (EC (European Commission), 2006). Table 3 shows some examples of PAH4 levels reported from the literature (between 2015 and 2020), far above the EU limit of 12 µg/kg (25 times (Iko Afé et al., 2020) or 52 times (Rozentale et al., 2018) this limit).

**TABLE 3:** Examples of levels of PAH4 above the maximal EU limit of 12 µg/kg in various smoked or grilled meat and fish

Products	PAH4 (µg/kg)	References
Smoked fish	198	Ingenbleek et al. (2019)
Smoked sprats	25.6	Gheorghe et al. (2019)
Grilled pork	53.8–300.6	Iko Afé et al. (2020)
*Slavonska kobasica*, smoked pork sausage	12.8–42.6	Mastanjević et al. (2020)
Smoked meat	56.2–628	Rozentale et al. (2018)
Smoked meat	34.6	Rozentale et al. (2015)
Barbecued pork	25.2	Duedahl‐Olesen et al. (2015)
Barbecued beef	48	

Note

PAH4: sum of benzo[a]pyrene, chrysene, benzo[b]fluoranthene, and benz[a]anthracene.

Determination of PAH in food can be performed after using an accelerated solvent extractor (ASE) for the extraction, and HPLC coupled with fluorescence and photo diode array detectors (FLD/PDA) or gas chromatography coupled with mass spectrometry (GC/MS) for quantification (Brasseur et al., 2007; Kendirci et al., 2014; Saito et al., 2014; Viegas et al., 2012).

During these past decades, several studies dealt with PAHs in processed food, especially in smoked meat and fish. Some of these studies reported in Tables 3 and 4 were from different continents: Asia (15.8%), Africa (42.1%), and Europe (42.1%). In Africa, Nigeria is the country in which more studies were carried out on PAHs. The PAHs data reported in Table 4 showed that most studies are recent (published between 2017 and 2021), showing that there is a new interest for scientists to update data on the presence of PAHs in smoked or grilled fish and meat. However, for countries such as Benin and Egypt, very few relevant data were available on PAHs contamination in fish and meat products before 2016 (Table 4). Most of the reported concentrations were above the EU maximal limit for BaP, and the highest BaP level (288 µg/kg) was about one hundred and forty‐four times above this limit, showing that consumers could be highly exposed to PAHs through the consumption of this kind of food.

**TABLE 4:** Examples of polycyclic aromatic hydrocarbon levels found in smoked or grilled fish and meat products in these past decades

Country	Type of food	Benzo(a)pyrene (µg/kg)	PAH4 (µg/kg)	References
Benin	Smoked *Scomber Scombrus*	5.6 ± 2.4	52.6 ± 20.4	Assogba et al. (2021)
	Smoked *Cypselurus cyanopterus*	23.0 ± 19.3	90.1 ± 93.3	
	Smoked‐dried *Cypselurus cyanopterus*	30.9 ± 16.2	153.8 ± 85.8 b	
	Grilled pork	28.9 ± 18.0	161.8 ± 87.2	Iko Afé et al. (2020)
	Smoked fish	21.8 ± 21.2	119.3 ± 107.5	Iko Afé et al. (2021)
	Smoked‐dried fish	78.5 ± 53.8	484.2 ± 305.6	
Croatia	Smoked sprat	2.2 ± 0.5	12.5 ± 1.9	Racovita et al. (2021)
Egypt	Grilled beef meat	2.7 ± 0.4	4.8 ± 0.9	Darwish al. (2019
	Grilled beef (kebab)	9.2	‐	Eldaly et al. (2016)
	Grilled beef (kofta)	26	‐	
Estonia	Smoked meat products	3.9	26.3	Rozentale et al. (2018)
France	Smoked *boucané* (pork product)	6.9 ± 2.4	‐	Poligné et al. (2001)
Ghana	Smoked Atlantic chub mackerel (*Scomber colias*)	15.5 ± 16.6	121.6 ± 98.9	Asamoah et al. (2021)
	Smoked barracuda (*Sphyraena Sphyraena*)	1.3 ± 2.1	68 ± 32.6	
Ivory Cost	Smoked *Cyprinus carpio*	16.9	‐	Ake Assi (2012)
	Smoked *Esox lucius*	56.5	‐	
	Smoked *Pagellus erythrinus*	36.7	‐	
	Smoked *Sarda* spp.	55.4	‐	
	Smoked *Sarpa salpa*	18.0	‐	
Korea	Charcoal broiled pork	2.6 ± 0.3	‐	Kim et al. (2014)
Kuwait	Meat tikka	2.5	‐	Alomirah et al. (2011)
Latvia	Smoked pork	35.1	‐	Stumpe‐Viksna et al. (2008)
	Smoked meat products	8.1	53.8	Rozentale et al. (2018)
Lithuania	Smoked meat products	1.9	9.5	Rozentale et al. (2018)
Nigeria	Smoked *Arius heude loti*	5.7	‐	Ubwa et al. (2015)
	Smoked Mud minnow	5.4	‐	
	Smoked *Scomber scombrus*	2.4	‐	Amos‐Tautua et al. (2013)
	Smoked *Clarias gariepinus*	204 ± 20	‐	Tongo et al., 2017; Zachara et al. (2017)
	Smoked *Ethmalosa fimbriata*	288 ± 230	‐	
	Smoked *Scomber scombrus*	7 ± 13	‐	
	Smoked *Pseudotolithus elongates*	44	‐	Akpan et al. (1994)
	Smoked *Pomadasys perotati*	25	‐	
	Smoked *Heterotis niloticus*	19.4	‐	
	Grilled suya*	10.1	‐	Akpambang et al. (2009)
	Grilled antelope*	7.9	‐	
	Smoked *Clarias gariepinus* *	38.0	‐	
	Smoked *Selar crumenophthalmus* *	3.0	‐	
	Smoked *Scomber scombrus* *	6.6	‐	
	Smoked *Pseudotolithus senegalensis* *	21.5	‐	
Poland	Smoked sprat	1	10.3	Zachara et al. (2017)
	Smoked sausage	3	24.3	
	Smoked pork hams	1.8	15.5	
Portugal	*Chouriço grosso*, dry‐cured fermented pork sausages*	3.3	‐	Roseiro et al. (2011)
	Grilled Salmon	4.7 ± 0.8	‐	Viegas, Novo, Pinto, et al. (2012)
	Chicken	8.7 ± 0.3	‐	
Spain	*Chorizo,* Spanish smoked pork meat	3.2	‐	Ledesma et al. (2015)
Turkey	Grilled anchovy fish (*Engraulis encrasicolus*)	0.7 ± 0.04	3.3 ± 0.1	Sahin et al. (2020)
	Grilled chicken	<LOD (0.05)	2.1 ± 0.1	

Abbreviations: ‐, data not presented in the cited paper; PAH4, sum of benzo[a]pyrene, chrysene, benzo[b]fluoranthene and benz[a]anthracene.

* Data of this author were presented in dry weight.

### 3.3 Other hazards in grilled or smoked fish and meat

#### 3.3.1 Heavy metals

Trace elements include environmental contaminants (heavy metals such as cadmium, mercury, and lead) which can have toxic effects on human health (Aina et al., 2012; Ismail et al., 2015) and oligo‐elements (copper, nickel, iron, cobalt, zinc, manganese, etc.) which play important physiological roles when they are at low concentrations. Heavy metals such as cadmium (Cd), mercury (Hg), and lead (Pb) are toxic even at low concentrations (Amos‐Tautua et al., 2013; Daniel et al., 2013; Ersoy et al., 2006; Şireli et al., 2006). Cadmium and arsenic are classified as carcinogenic for humans (Group 1) and lead is classified as possibly carcinogenic for humans (Group 2B) by the IARC (International Agency for Research on Cancer) (2010). Environmental pollution is the main way of food contamination with heavy metals (Costa et al., 2016; EFSA (European Food Safety Authority), 2010). In 2010, the European Food Safety Authority reported that human exposure to lead through diet results in its bioaccumulation responsible for adverse effects on the cardiovascular, renal, endocrine, gastrointestinal, immune, and reproductive systems. The Codex Alimentarius reported that lead was responsible for the low intellectual quotient based on lead exposure studies in children (Codex Alimentarius, 2004). European Commission set a maximum limit of 0.1 mg/kg for lead in meat (excluding offal) of bovine animals, sheep, pig, and poultry and 0.3 mg/kg in muscle of fish (EC (European Commission), 2006). For cadmium, the maximal limit is 0.1 mg/kg in muscle of mackerel (*Scomber* spp.), tuna (*Thunnus* spp., *Katsuwonus pelamis*, *Euthynnus* spp.), and bichique (*Sicyopterus lagocephalus*), whereas in meat products, the maximal limits range between 0.05 and 1 mg/kg, depending on the species and the tissue of the animal.

Several authors reported the presence of trace elements in smoked fish (Anigboro et al., 2011; Ibanga et al., 2019; Inobeme et al., 2018). Şireli et al. (2006) reported the presence of lead (0.01–0.8 mg/kg) in vacuum packaged smoked fish marketed on the Ankara market in Turkey (Table 5). In that study, 37% of the smoked fish samples were not compliant to the Turkish acceptable limit of 0.2 mg/kg. Likewise, Anigboro et al. (2011) reported high levels of lead (13–59 mg/kg) in smoked fish samples (Table 5) collected from different local markets in Nigeria. Arsenic was found in smoked *Dicentrarchus labrax* (0.4 mg/kg), *Scomber scombrus* (0.4 mg/kg), *Clarias gariepinus* (0.02 mg/kg), and *Ethmalosa fimbriata* (0.02 mg/kg) (Table 5). For cadmium, examples of concentration reported from the literature are shown in Table 5.

**TABLE 5:** Mean concentrations of heavy metals in smoked or grilled fish (a) and meat (b) products as reported from the literature

(a)								
Country	Fish species	Heavy metals (mg/kg)						References
		Pb	Cd	Hg	Ni	As	Cr	
Egypt	*Ctenopharyngodon idella*	nd	0.2	nd	7.7	nd	nd	Abbas et al. (2021)*
Iran	*Rutilus frissi*	0.003	0.002	nd	nd	nd	0.002	Mehdipour et al. (2018)*
Nigeria	*Scomber scombrus*	nd	nd	nd	nd	0.40	0.1	Aremu et al. (2014)
	*Clarias gariepinus*	0.2	2.5	0.02	12.8	0.02	nd	Ibanga et al. (2019)*
	*Ethmalosa fimbriata*	0.2	19.5	0.02	12.4	0.02	nd	
	*Heteroclaria*	18.7	1	nd	123.3	nd	50.3	Anigboro et al. (2011)
	*Ethmalosa fimbriata*	21.3	2.2	nd	120.7	nd	54.3	
	*Tilapia guineensis*	43.7	nd	nd	148.7	nd	71	
Poland	Herring	0.04	0.004	nd	nd	nd	nd	Rajkowska‐Myśliwiec et al. (2021)
	Sprats	0.02	0.02	nd	nd	nd	nd	
Spain	Sardine	0.04	0.002	0.03		3.3	nd	Perello et al. (2008)
	Hake	0.02	nd	0.2		1.4	nd	
	Tuna	0.03	0.002	0.4		1.6	nd	
Turkey	*Dicentrarchus labrax*	0.3	nd	nd	0.2	0.4	0.05	Ersoy et al. (2006)
	*Salmo salar*	0.2	0.02	nd	nd	nd	nd	Şireli et al. (2006)*
	*Oncorhynhus mykiss*	0.1	0.01	nd	nd	nd	nd	
	Mackerel	0.05	0.01	nd	nd	nd	nd	
	*Oncorhynhus mykiss*	0.4	0.02	nd	nd	nd	nd	

(b)								
Country	Meat products	Heavy metals (mg/kg)						References
		Pb	Cd	Hg	Ni	As	Cr	
Burkina‐Faso		Braised chicken	0.2	0.5	nd	nd	Nd	Bazié et al. (2021)
		Flamed chicken	0.1	0.2	nd	nd	Nd	
Ghana	Bush meat	3.6	0.1	nd	nd	nd	nd	Kobia et al. (2016)
Spain	Veal steak	0.02	nd	nd	nd	0.2	nd	Perello et al. (2008)
	Loin pork	nd	nd	nd	nd	0.2	nd	
	Chicken	nd	nd	nd	nd	0.1	nd	
	Lamb	nd	nd	nd	nd	0.2	nd	

Abbreviation: nd, not determined.

* Reported data were expressed in dry matter.

Perello et al. (2008) reported the increase of Pb, As, and Hg contents in fish and meat products processed with grilling, frying, boiling, and roasting, compared to the raw products collected from Spain markets (data not shown). Even though an increase of heavy metal levels was recorded after processing in different studies, this increase could be due to the absorption phenomenon or environmental contamination as the culinary practices were not carried out in controlled close space. It could also be a concentration of the contaminants due to water loss during smoking and drying.

Heavy metal concentrations can be measured by a graphite furnace atomic absorption spectrometer (GFAAS) or an atomic absorption spectrophotometer (Anigboro et al., 2011; Şireli et al., 2006). They could also be determined using atomic absorption spectrometry after microwave digestion and inductively coupled plasma mass spectrometry (ICP/MS) (Kabir et al., 2011; Uluozlu et al., 2009). The studies on the occurrence of heavy metals in smoked or grilled fish and meat reported in this section were mainly from Africa. Indeed, although some studies were from Turkey (Ersoy et al., 2006; Şireli et al., 2006), Spain (Perello et al., 2008) and Poland (Rajkowska‐Myśliwiec et al., 2021), the majority of them were from Nigeria (Amos‐Tautua et al., 2013; Anigboro et al., 2011; Aremu et al., 2014; Daniel et al., 2013; Ersoy et al., 2006; Ibanga et al., 2019) and other African countries such as Egypt (Abbas et al., 2021), Ghana (Kobia et al., 2016) and Burkina Faso (Bazié et al., 2021). Heavy metals contamination data (Table 5) showed that before 2015 (2006–2014) many studies from different countries especially Turkey and Nigeria were carried out on the occurrence of these environmental contaminants in smoked or grilled fish and meat. From 2015 to 2021, additional studies from Nigeria were carried out again on these compounds showing the necessity to update contamination data in smoked or grilled fish and meat. However, for other countries such as Burkina‐Faso or Egypt, very few relevant data were available on heavy metals contamination in smoked or grilled fish and meat before 2015 (Table 5).

#### 3.3.2 Biogenic amines

Biogenic amines are found in protein‐rich foods such as fish and meat products (Chong et al., 2011; Latorre‐Moratalla et al., 2017; Sagratini et al., 2012). Despite the important role of some biogenic amines in human and animal physiology, the consumption of a high amount of these amines can result in food intoxication (EFSA (European Food Safety Authority), 2011; Lehane & Olley, 2000). They are usually produced from decarboxylation of free amino acids by bacterial enzymes (Table 6), before or after processing. They are also heat resistant, so not destroyed by the cooking practices. Among biogenic amines, histamine received particular attention due to its toxicity. Several authors reported histamine as responsible for foodborne intoxication in reference to scombroid fish poisoning (EFSA (European Food Safety Authority), 2011; Latorre‐Moratalla et al., 2017). Intoxication with histamine is associated with symptoms such as hypertension, headache, and allergy reactions including reddening on the face, neck and upper chest, vomiting, sweating, nausea, abdominal cramps, diarrhea, rash, dizziness, palpitations, spasm of bronchi, and flushing (Hassan, El‐ Shater, & Waly, 2017; Marissiaux et al., 2018; da Silva, Pinho, Ferreira, Plestilova, & Gibbs, 2002; Zaman et al., 2010). Several papers reported biogenic amines in smoked fish and grilled meat products (Douny et al., 2019; Köse et al., 2012; Ntzimani et al., 2008; Simunovic et al., 2019). The histamine concentrations reported by several authors in these kinds of food are summarized in Table 7. The presence of histamine was reported in smoked salmon at levels ranging between 2.5 and 171 mg/kg, in smoked *Sardinella* sp. (18 mg/kg), and in hot smoked bonito (98.7 ± 0.6 mg/kg) (Table 7). The presence of histamine was also reported in grilled pork (<11.2–81.5 mg/kg) and in smoked turkey (32.9 ± 1.4 mg/kg) (Table 7).

**TABLE 6:** Structure, precursors, and microorganisms producing decarboxylase of some biogenic amines

Amino acid precursors	Biogenic amine	Chemical structure and formula	Main microorganisms producing amino acid decarboxylase
Histidine	Histamine	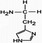 C_5_H_9_N_3_	*Hafnia alvei, Morganella morganii, Klebsiella pneumonia, Morganella psychrotolerans,* *Photobacterium phosphoreum,* *Photobacterium psychrotolerans*
Tryptophan	Tryptamine	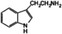 C_10_H_12_N_2_	‐
Tyrosine	Tyramine	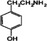 C_8_H_11_NO	*Enterococcus* (*Ent. faecalis, Ent. faecium*) *Lactobacillus* (*Lact. curvatus; Lact. brevis*) *Leuconostoc* spp, *Carnobacterium* spp *Staphylococcus* spp
Phenylalanine	2‐Phenylethylamine	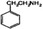 C_8_H_11_N	Enterococcus, *Lactobacillus curvatus*, Staphylococcus (*S. carnosus*)
Hydroxytryptophan	Serotonine	nd	‐
Lysine	Cadavérine	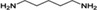 NH_2_(CH_2_)_5_NH_2_	*Enterobacteriaceae* (*Citrobacter*, *Klebsiella*, *Escherichia*, *Proteus*, *Salmonella* et *Shigella)* *Pseudomonadaceae, Shewanellaceae*
Ornithine; arginine	Putrescine	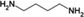 NH_2_(CH_2_)_4_NH_2_	*Enterobacteriaceae* (*Citrobacter*, *Klebsiella*, *Escherichia*, *Proteus*, *Salmonella* et *Shigella)* *Pseudomonadaceae, Shewanellaceae*
Ornithine; arginine	Spermine	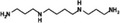 C_10_H_26_N_4_	‐
Ornithine; arginine	Spermidine	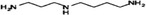 C_7_H_19_N_3_	‐

**TABLE 7:** Histamine levels in smoked or grilled fish and meat products

Product	Concentration (mg/kg)	Analytical method	References
Smoked salmon	2.5–171	Extraction with Trichloroacetic acid; LC‐MS/MS	Simunovic et al. (2019)
Smoked *Sardinella* sp.	18	Extraction trichloroacetic acid ion‐exchange chromatography	Plahar et al. (1999)
Cold‐smoked salmon	30.9 ± 0.4	Extraction with perchloric acid high‐performance liquid chromatography with a diode array detector	Köse et al. (2012)
Hot‐smoked Bonito (Tuna fish)	98.7 ± 0.6		
Grilled tuna	4,400	Not mentioned	Marissiaux et al. (2018)
Smoked fish from different species	11–63	Quantification colorimetrically at 495 nm using a spectrophotometer.	CSIR (2017)
Smoked turkey breast fillets stored at 4°C after 30 days	32.9 ± 1.4	Extraction trichloroacetic acid With liquid chromatography. Quantification was performed coupled with a UV detector	Ntzimani et al. (2008)
Grilled pork	<11.2–81.5	Extraction with perchloric acid and injection on UPLC coupled with a fluorescence detector	Douny et al. (2019)

European Commission set maximal limits for histamine (100–200 mg/kg) in fish and fishery products from fish species associated with a high amount of histidine (EC (European Commission), 2005). No maximal limit of histamine is available for meat products. However, several authors reported the use of biogenic index (sum of putrescine, tyramine, cadaverine, and histamine levels) to assess the freshness and quality of pork (Cheng et al., 2016; Douny et al., 2019).

The highest histamine concentration in fish reported in this review was 44 times over the authorized European limit and resulted in histamine fish poisoning (HFP) (Marissiaux et al., 2018). Similar concentration (4,384.2 mg/kg) was also reported in smoked‐dried fish from Benin (Table 8). Regarding the geographical location, the selected paper reported in the Tables 7 and 8 was from America (5%), Asia (20%), Africa (30%), and Europe (45%). Although several studies dealt with the production of biogenic amines in fish, very few studies were available about grilled and/or smoked fish and meat products. From 2015 to 2021, studies dealing with biogenic amines in grilled or smoked fish and meat mainly focused on their occurrence (Tables 7 and 8).

**TABLE 8:** Mean (maximum) concentrations of histamine and tyramine in fish (a) and meat products (b) from different countries

(a)				
Country	Type of food	Histamine (mg/kg)	Tyramine (mg/kg)	References
Austria	Smoked tuna	‐ (63)	‐	Rauscher‐Gabernig et al. (2009)
	Smoked mackerel	‐ (219)	‐	
	Smoked salmon	‐ (165)	‐	
Belgium	Grilled tuna fish	‐ (4,400)	‐	Marissiaux et al. (2018)
Benin	Smoked *Cypselurus cyanopterus*	471.7 (1,139.4)	810.9 (1766.5)	Assogba et al. (2021)
	Smoked‐dried *Cypselurus cyanopterus*	754.3 (2,255.1)	19.1 (20.6)	
	Smoked fish	<10 (1,511.3)	151.9 (700.9)	Iko Afé et al. (2021)
	Smoked‐dried fish	1,340.2 (4,384.2)	33.1 (45.8)	
Cambodia	Smoked fish	16.6 (24.2)	9.9 (38.4)	Douny et al. (2021)
Denmark	Cold‐smoked tuna	4,548 (‐)	150 (‐)	Emborg and Dalgaard (2006)

(b)				
Country	Type of food	Histamine (mg/kg)	Tyramine (mg/kg)	References
Benin	Grilled pork	59.7 (86.4)	2 (3.8)	Douny et al. (2019)
Egypt	Beef shawarma	80.2 (30)	‐	Sallam et al. (2021)
	Chicken shawarma	103 (36)	‐	
Spain	Dry fermented sausages	27 (475)	139 (742)	Latorre‐Moratalla et al. (2017)

Abbreviation: ‐, data not presented in the cited paper.

* Numbers in parentheses represent the maximum value.

### 3.4 Risk assessment

#### 3.4.1 Risk assessment methodology applicable to toxic compounds

The risk assessment is part of the risk analysis concept, which, as reported by Larsen (2006), includes risk assessment, risk evaluation, and risk communication. These three elements are separate tasks, performed by different actors, but should be part of an interactive process (Larsen, 2006; Stadler & Lineback, 2009). Risk assessment is a scientific process used to quantify the risk linked to a hazard and requires expertise in toxicology and nutrition (for the intake assessment). It is used to determine whether a particular chemical poses a significant risk to human health (FASFC (Federal Agency for the Safety of the Food Chain), 2005; Larsen, 2006; Reinik, 2007; Stadler & Lineback, 2009; Scholl et al., 2012). Risk assessment follows four steps (EFSA (European Food Safety Authority), 2008; FASFC (Federal Agency for the Safety of the Food Chain), 2005; FASFC (Federal Agency for the Safety of the Food Chain), 2006; Larsen, 2006) which are as follows:

***Hazard identification***: It will indicate which dangers can be associated with the consumption of a specific foodstuff and what harmful effects they can cause for consumers.

***Hazard characterization***: This step aims to describe and evaluate the dose–response relationship, the mode of action, including dynamic and kinetic aspects, and how to establish an acceptable daily intake (ADI) or a tolerable daily intake (TDI) using a safety factor to consider for the intra‐ and inter‐species variation.

***Exposure assessment***: To assess the exposure, consumption data and contamination data are needed to calculate the estimated daily intake (EDI) by multiplying the concentration of hazard by the daily consumption of food contaminated with this hazard. EDI can be calculated for several categories of population (i.e., babies, children, teenager, and adults). EDI can be calculated either following a deterministic approach using median, mean, or maximum of consumption or contamination data, or following a probabilistic approach using distributions of consumption and contamination data.

***Risk characterization***: This step consists of comparing the calculated EDI with a toxicological reference dose (classical way) which can be a tolerable daily intake (TDI) or an acceptable daily intake (ADI). For carcinogenic compounds such as PAHs, the margin of exposure (MoE) suggested by EFSA (European Food Safety Authority) (2005) and Constable and Barlow (2009) is used. MOE is calculated as follows:

$$\mathrm{MOE}=\frac{\mathrm{BMDL}10\;(\mathrm{mg}\;\mathrm{per}\;\mathrm{kg}\;\mathrm{bw}\;\mathrm{per}\;\mathrm{day})}{\mathrm{EDI}\;(\mathrm{mg}\;\mathrm{per}\;\mathrm{kg}\;\mathrm{bw}\;\mathrm{per}\;\mathrm{day})},$$

where BMDL_10_ is the 95% lower confidence limit of the benchmark dose causing 10% extra risk of cancer in laboratory animals (in case of PAHs, of rat hepatocellular adenomas, and carcinoma), and EDI is the estimated daily intake. For carcinogenic compounds such as PAHs, the risk may be considered as negligible or very low only when MOE is above 10,000.

#### 3.4.2 Examples reported from the literature of risk assessment for some chemical hazards (PAHs, heavy metals, and biogenic amines)

Examples of risk assessments related to PAH ingestion through consumption of grilled and/or smoked fish and meat (Table 9) pointed out a health concern for consumers of several countries such as Cambodia (Douny et al., 2021), Benin (Iko Afé et al., 2020, 2021), Turkey (Sahin et al., 2020), Nigeria (Akpambang et al., 2009), and Latvia (Rozentale et al., 2018). Before 2015, the mean values of MoE associated with the consumption of smoked or grilled fish (Table 9a) and meat products (Table 9b) contaminated with PAHs including BaP and PAH4 were globally above 10,000, showing a very low concern for the consumers of these products. After 2015, the studies reported showed MoE globally below 10,000 for consumers of smoked or grilled fish and meat products from different countries such as Benin, Cambodia, Turkey, and Latvia (Table 9). MOE below 10,000 indicates a high concern (risk of cancer) for consumers for carcinogenic compounds such as PAHs.

**TABLE 9:** Estimated daily intakes (EDI) and margin of exposure (MOE) for polycyclic aromatic hydrocarbons (PAH) through consumption of smoked or grilled fish (a) and meat (b) products, in different countries

(a)				
Country	Type of food	Estimated daily intake (ng/kg bw/day)	Margin of exposure	References
Benin	Smoked fish	BaP: 2.3‐809.9	BaP: 30,978‐86	Iko Afé et al. (2021)
		PAH4: 12.0‐4,314.9	PAH4: 28,241‐79	
	Smoked‐dried fish	BaP: 2.5‐1,974.8	BaP: 27,718‐35	
		PAH4: 17.4‐13,627.2	PAH4: 19,510‐25	
Cambodia	Smoked fish	BaP: 1,407	BaP: 50	Douny et al. (2021)
		PAH4: 5,773	PAH4: 59	
China	Grilled fish	BaP: 0.2	BaP: 333,000	Wang et al. (2021)
		PAH4: 1.0	PAH4: 336,000	
Croatia	Shellfish products	BaP: ‐	BaP: 1,643,906	Bogdanovic et al. (2019)
		PAH4: ‐	PAH4: 298,900	
Nigeria	Smoked fish	BaP: 4‐52	BaP: 17,722‐1,346	Akpambang et al. (2009)
		PAH4: ‐	PAH4: ‐	
Turkey	Grilled fish	BaP: 0.2	BaP: 389	Sahin et al. (2020)
		PAH4: 0.8	PAH4: 425	

(b)				
Country	Type of food	Estimated daily intake (ng/kg bw/day)	Margin of exposure	References
Benin	Grilled pork	BaP: 07‐235.7	BaP: 96,871‐297	Iko Afé et al. (2020)
		PAH4: 4.1‐1,321.3	PAH4: 83,925‐257	
China	Grilled meat	BaP: 0.5	BaP: ‐	Jiang et al. (2018)
		PAH4: 4.0	PAH4: ‐	
Croatia	Smoked meat products	BaP: ‐	BaP: 280,657	Bogdanovic et al. (2019)
		PAH4: ‐	PAH4: 66,213	
Denmark	Home grilled meat (beef, pork, and chicken)	BaP: ‐	BaP: ‐	Duedahl‐Olesen et al. (2015)
		PAH4: 10	PAH4: 33,800	
Egypt	Grilled beef meat	BaP: 290.5	BaP: ‐	Darwish al. (2019
		PAH4: ‐	PAH4: ‐	
France	Foodstuffs (28 different foods)	BaP: 0.2	BaP: ‐	Veyrand et al. (2013)
		PAH4: 1.5	PAH4: 230,000	
Korea	Smoked meat products (bacon, chicken, duck, pork, salmon, tuna, and turkey).	BaP: 0.014	BaP: 666,667	Kim et al. (2014)
		PAH4: 0.038	PAH4: 265,957	
Kuwait	Grilled chicken	BaP: 15.6	BaP: ‐	Alomirah et al. (2011)
		PAH4: ‐	PAH4: ‐	
Latvia	Smoked meat products	BaP: 5.4	BaP: 12,952	Rozentale et al. (2018)
		PAH4: 35.9	PAH4: 9,475	
	Smoked meat (pork, pork breast, chop, speck, ham, and chicken) and meat products (sausages, small sausages, semi‐dry sausages, and roulette)	BaP: 2.3	BaP: 30,606	Rozentale et al. (2015)
		PAH4: 13.7	PAH4: 24,776	
Nigeria	Grilled meat	BaP: 10.5‐14.0	BaP: 5,015‐6,652	Akpambang et al. (2009)
		PAH4: ‐	PAH4: ‐	
Turkey	Grilled chicken	BaP: ‐	BaP: ‐	Sahin et al. (2020)
		PAH4: 1.8	PAH4: 190	

Abbreviations: ‐, data not presented in the cited paper; PAH4, sum of benzo[a]pyrene, chrysene, benzo[b]fluoranthene, and benz[a]anthracene; bw, body weight.

Regarding consumers exposure to heavy metals from consumption of smoked or grilled fish and meat products, very few data were available from the literature. Recently, two papers have been published on exposure of consumers from Burkina‐Faso (Bazié et al., 2021) and Poland (Rajkowska‐Myśliwiec et al., 2021).

The cancer risk index linked to lead exposure calculated for consumers of braised and flamed chicken processed in Burkina Faso ranged between 7 × 10^−7^ and 3 × 10^−6^ (Table 10). None of the index risk values was above the threshold set by US‐EPA (IR > 10^−4^). Similar to the cancer risk index, a noncancer risk index was calculated using the median consumption level of braised and flamed chicken. This Hazard Index (HI), which is the sum of individual metal hazard (Ag, Cd, Pb, Zn, Ni, Co, Fe, Mn, Cu, and Cr) quotients, ranged between 0.07 and 0.15. These values were below the reference value (HI = 1) (Hough et al., 2004) showing also the absence of noncancer risk linked to heavy metals exposure for Burkina‐Faso consumers of braised and flamed chicken (Bazié et al., 2021). However, the HI (sum of hazard quotient of Zn, Fe, Mn, Cu, Al, Pb, and Cd) calculated for polish consumers was 1.4 (Table 10), so above the reference value of 1. The HI obtained for polish consumers was similar to HI values reported for Ugandan consumers of heat‐processed meat which ranged from 1.2 to 1.9 for different types of meats (Table 10).

**TABLE 10:** Cancer and noncancer risks related to heavy metals through consumption of smoked or grilled fish and meat products reported from the literature

Country	Type of food	Noncancer risk: Hazard index (HI)	Cancer index risk (IR)	References
Burkina‐Faso	Flamed chicken	0.2	Pb: 7 × 10^−7^ to 3 × 10^−6^	Bazié et al. (2021)
	Braised chicken	0.1	Pb: 7 × 10^−7^ to 3 × 10^−6^	
Poland	Smoked fish	1.4	‐	Rajkowska‐Myśliwiec et al. (2021)
Uganda	Roasted pork	1.7	Pb: 4.5 × 10^–5^	Bamuwamye et al. (2015)
			Cd: 1.0 × 10^–3^	
			As: 7.4 × 10^–5^	
	Roasted beef	1.7	Pb: 3.92 × 10^–5^	
			Cd: 6.30 × 10^–4^	
			As: 2.00 × 10^–4^	
	Roasted goat	1.2	Pb: 2.95 × 10^–6^	
			Cd: 2.60 × 10^–3^	
			As: 9.94 × 10^–5^	
	Roasted chicken	1.9	Pb: 2.50 × 10^–5^	
			Cd: 2.00 × 10^–3^	
			As: 3.00 × 10^–4^	

Abbreviation: ‐, data not presented in the cited paper.

Among biogenic amines, histamine and tyramine are two dietary biogenic amines which are present in food are undesirable due to their adverse effects on consumer's health such as hypertension, headache, and allergic reactions (EFSA, 2011; Marissiaux et al., 2018). To our best knowledge, there are few relevant studies showing the exposure to histamine or tyramine for consumers of smoked or grilled fish and meat products. Four studies dealing with histamine exposure were reported in Table 11. These four studies were published during the period of 2017–2021. The mean histamine intake calculated from the consumption of smoked fish and smoked‐dried fish marketed in Benin was 146 mg/meal and 116 mg/meal, respectively, whereas the acute reference dose (ARfD) of histamine suggested by the European Food Safety Authority (EFSA) is 50 mg histamine/meal (EFSA, 2011). In Spain, Cambodia, and Egypt, the mean histamine exposure (Table 11) was well below this ARfD. Based on the limited published data, no adverse health effects have been observed in healthy volunteers exposed to a level of 25–50 mg of histamine per person per meal (EFSA, 2011). The mean histamine exposure reported in Table 11 revealed a health concern for Beninese consumers of smoked fish and smoked‐dried fish (Iko Afé et al., 2021). Although the mean histamine exposure reported for consumers of Cambodia, Spain, and Egypt showed an absence of intoxication risk, there is risk of histamine poisoning in case of extreme consumption of smoked or grilled fish and meat products during the same meal or for sensitive consumers.

**TABLE 11:** Histamine and tyramine exposure from consumption of fish and meat products

Country	Type of food	Histamine exposure (mg/meal)	Tyramine exposure (mg/meal)	References
Benin	Smoked fish	145.6 (1,019.1)*	‐	Iko Afé et al. (2021)
	Smoked‐dried fish	115.9 (1,236.2)	‐	
Cambodia	Smoked fish	<50 (‐)	‐	Douny et al. (2021)
Egypt	Beef shawarma	16.0	‐	Sallam et al. (2021)
	Chicken shawarma	31	‐	
Spain	Dry fermented sausages	1.4 (45.8)	6.2 (92.5)	Latorre‐Moratalla et al. (2017)

Abbreviation: ‐, data not presented in the cited paper.

* Numbers in parentheses represent the maximum value.

## 4 CONCLUSION

Smoked fish and meat products may be contaminated by various toxic compounds including carcinogenic compounds. Most of the chemical hazards reported in this review are processing contaminants. Some of them can be formed when high temperature is reached inside the product (heterocyclic amines and nitrosamines) and others during pyrolysis of the fuel during processing (PAHs). Biogenic amines are not related to the smoking process but can be present in raw or smoked fish due to decarboxylation of free amino acids occurring after microbial contamination. In case of heavy metals, they are environmental pollutants found in raw and processed food. In traditionally smoked fish or grilled meat, most of the chemical hazards mentioned in this review exceed the maximal limits established by EU. Several actions should be addressed to decrease them in smoked fish and meat as they are highly consumed products.

## CONFLICT OF INTEREST

The authors have no conflicts of interest to declare.

## ETHICAL APPROVAL

This study does not involve any human or animal testing.

## Data Availability

All the data used in this study can be made available upon reasonable request.
